# Large-scale paired chain BCR analysis reveals antibody clonal family inference bias and enhances resolution with machine learning

**DOI:** 10.1371/journal.pcbi.1014077

**Published:** 2026-03-11

**Authors:** Hao Wang, Kaixuan Wang, Qihang Xu, Linru Cai, Chuanxiang Huang, Linlin Chen, Yunliang Zang, Xihao Hu, Jian Zhang

**Affiliations:** 1 Academy of Medical Engineering and Translational Medicine, Tianjin University, Tianjin, China; 2 GV20 Therapeutics, Cambridge, Massachusetts, United States of America,; 3 Key Laboratory of Systems Bioengineering (Ministry of Education), Tianjin University, Tianjin, China; University of Louisville, UNITED STATES OF AMERICA

## Abstract

A fundamental question in immunology is how the adaptive immune system encodes antigen specificity while maintaining repertoire diversity. B cell receptor (BCR) or antibody clonal families, defined by groups of B cells descending from a common ancestor, are key to deciphering this encoding. Although paired heavy and light chains jointly determine antibody specificity, most repertoire analyses have historically relied on heavy-chain-only data due to the loss of native pairing information in bulk BCR sequencing. This reliance introduces potential biases in computational clonal cluster inference, which may complicate efforts to resolve disease-associated immune signatures. Here, we leverage large-scale paired-chain BCR sequencing data to demonstrate that heavy-chain-based clustering may misrepresent true clonal architecture, and identify two major artifacts: chain-mixed clusters, in which similar heavy chains are paired with distinct light chains, and naive-like pseudo-clonal clusters, which are detected in an individual’s naive B cell repertoire and exhibit highly similar heavy and light chains without reflecting true clonal expansion. To address these limitations, we present fastBCR-p, an optimized framework that integrates light-chain-informed subclustering, with public sequence aware refinement to improve clonal family inference. By resolving both technical artifacts and biological convergence, fastBCR-p improves the chain concordance and overall clustering quality of clonal inference in real-world datasets. This enables more accurate tracking of immune dynamics in health and disease and facilitates the identification of clinically relevant antibody lineages.

## Author summary

Our immune system protects us by producing a vast and diverse collection of antibodies, each designed to recognize a specific target. These antibodies are made by B cells, which expand and evolve in groups known as clonal families. Accurately identifying these clonal families from sequencing data is essential for understanding immune responses during infection, vaccination, and disease. Most existing computational methods infer B-cell clonal families using information from only one part of the antibody, the heavy chain. This limitation largely reflects the fact that traditional sequencing technologies often lose information about how heavy and light chains are naturally paired. However, both chains are required to define antibody specificity. Using large-scale datasets that preserve native heavy–light chain pairing, we show that heavy-chain-only approaches can introduce systematic errors. These include incorrectly grouping together unrelated B cells and falsely identifying naive B cells as expanded clones. To address these limitations, we developed fastBCR-p, which integrates light-chain information and accounts for shared (“public”) antibody sequences. By correcting both technical artifacts and biological convergence, fastBCR-p enables more accurate clonal family inference and improves the analysis of immune repertoire dynamics and facilitating the identification of clinically relevant antibody lineages.

## Introduction

The adaptive immune system uses a diverse repertoire of B cell receptors (BCRs) to recognize and eliminate a wide range of pathogens and abnormal cells. During maturation in the bone marrow, individual B cells undergo V(D)J gene rearrangements [1] that are often modeled as stochastic processes, along with probabilistic pairing of heavy-chains (HC) and light-chains (LC) [2], ultimately creating a unique BCR. Although accumulating evidence indicates that these processes are not purely random and can be influenced by locus architecture and inter-individual germline variation [3,4], they nevertheless enable the generation of enormous diversity. Theoretically, such approximate random rearrangements can generate up to ${\text{1}\text{0}}^{\text{1}\text{8}}$ possible BCRs [5], while an individual’s repertoire may contain as many as ${\text{1}\text{0}}^{\text{1}\text{2}}$ distinct clonotypes [6]. Moreover, upon antigen exposure, B cells undergo clonal expansion and accumulate somatic hypermutations (SHM) [7] in the complementarity-determining regions (CDRs) of their BCRs. This process gives rise to families of B cells sharing a common ancestor [8] and featuring highly similar BCR sequences, thereby enabling progressive affinity maturation toward a given antigen [9–11]. Following multiple rounds of in vivo selection, high-affinity B cells differentiate into plasma cells that secrete antigen specific antibodies, while a subset is maintained as memory B cells, ensuring rapid recall responses upon re-encounter with the same or similar antigens. Accordingly, delineating the affinity maturation process is pivotal not only for understanding how the immune system recognizes and combats foreign antigens but also for advancing antibody drug discovery [12,13], various disease research [14–18], and vaccine design [19,20].

Despite substantial progress, accurately identifying B cell clonal families triggered by antigen stimulation remains challenging. High-throughput BCR sequencing (BCR-seq) [21] can comprehensively capture the BCR repertoire in each sample [22–24], thus offering an opportunity to infer clonal family information. However, most widely utilized bulk BCR-seq protocols [25]—although capable of generating large amounts of HC and LC sequence data—do not readily preserve the pairing information between individual HC and LC. Consequently, existing computational approaches typically focus on HC data to identify B cell clonal families [26–29]. In practice, these methods stratify sequences by V/J gene usage, cluster on junctional similarity using Hamming/Levenshtein or k-mer distances with SHM-aware thresholds, and in some cases refine clades via likelihood-based lineage reconstruction. Indeed, the HC has been shown to exhibit higher sequence variability [30] and often plays a critical role in antigen recognition [31], making HC-based approaches efficient and broadly applicable [32,33]. Nevertheless, the HC-based strategy may fail to capture the full clonal architecture, especially when selective forces shape the repertoire through LC-biased or convergent pairing events.

Critically, the foundational assumption that HC similarity alone reliably reflects true clonal structure has not been systematically evaluated at large scale [29]. With the increasing availability of paired-chain datasets, it is now possible to directly test this assumption. Early analyses suggest that omitting LC information can lead to substantial misclustering and artificial convergence of unrelated B cells [34,35]. These artifacts are especially problematic when studying immune repertoires in disease contexts, where identifying lineage-specific or antigen-selected BCRs is of biological and clinical relevance. Furthermore, recent studies have begun to explore convergent immune features—such as recurrent BCR motifs—across individuals and disease states [36,37], raising the possibility of conserved humoral responses. However, identifying such shared clonotypes is complicated by single-chain inference artifacts, which may exaggerate sequence sharing due to public sequence generation biases rather than true biological convergence.

To address these limitations, we developed a data-driven framework to assess how well HC-only methods capture true clonal family structure, using ~2 million naturally paired BCR sequences from 218 peripheral blood samples in the Observed Antibody Space (OAS) database [38] as a benchmark. This analysis revealed that, particularly in immunologically complex settings, HC similarity alone may produce clonal groupings that lack coherent biological signatures, including chain discordance and the aggregation of convergent, naive-like sequences. These systematic evaluation results motivate the need for refinement strategies that leverage paired-chain information and explicitly account for biologically driven sequence convergence. We therefore introduce fastBCR-p as a practical framework that incorporates paired chain aware subclustering and public sequence aware refinement to address these failure modes, enabling more faithful reconstruction of clonal relationships from large-scale repertoire datasets and more reliable analysis of B cell dynamics.

## Results

### Reconstructing clonotype structure from paired-chain repertoires

**Fig 1:**
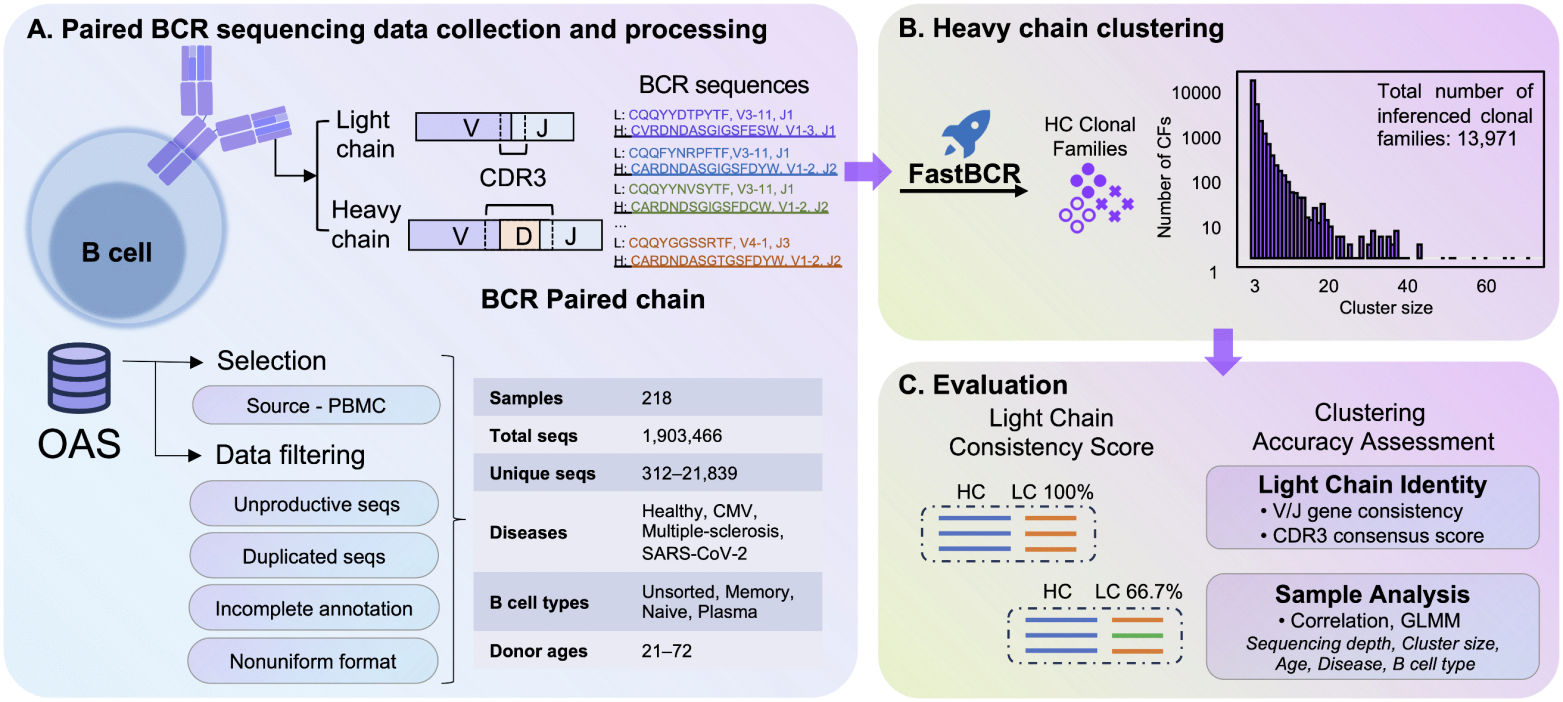
Schematic overview of the analysis workflow. (A) Paired BCR sequencing samples were downloaded from the OAS database. After data filtering, high-quality paired BCR sequences were retained, covering multiple disease and B cell subtypes. (B) Heavy-chain (HC) clustering performed by *fastBCR*, with the bar plot showing the size distribution of the resulting clonal families. (C) Evaluation of light-chain (LC) consistency within each HC-derived cluster, including V/J gene usage and CDR3 similarity scores, to validate clustering accuracy and paired-chain integrity.

To investigate how partial observations of the BCR repertoire distort our understanding of immune architecture, we first established a framework for quantifying the fidelity of HC-based clonotyping relative to ground-truth paired-chain data. This framework encompasses the collection and preprocessing of paired-chain BCR data, HC-based clonal clustering, and subsequent in-depth assessments of LC concordance within each cluster (Fig 1).

First, we downloaded the paired HC and LC human BCR sequences from the OAS database [38], encompassing seven independent research projects (S1 Table). During preprocessing, we selected only peripheral blood samples and removed sequences that contained missing annotations or exhibited low sequencing coverage. This ensured that every retained sequence was paired, high-quality, and fully annotated for both V, J genes and the complete CDR3 region. Next, to minimize bias arising from redundant entries, we removed identical amino acid sequences appearing within the same sample or the same donor, retaining only one representative for each duplicated sequence. Following these procedures, we obtained a final dataset consisting of 1,903,466 high-quality paired-chain sequences derived from 218 individual samples. The sequencing depths varied from 312 to 21,839 unique sequences per sample. These samples encompassed four distinct conditions: Cytomegalovirus (CMV), Multiple Sclerosis, SARS-CoV-2, and Healthy controls. Additionally, the dataset included four B cell subtypes: Unsorted B cells, Memory B cells, Naive B cells, and Plasma cells. The ages of the donors ranged from 21 to 72 years, allowing for a comprehensive analysis across a broad spectrum of biological conditions (Fig 1A).

Next, we employed our previously published tool recognized for its computational efficiency, *fastBCR* [39,40], to cluster HC sequences within each sample. Without filtering for low sequence consistency, and excluding singletons and doubletons, *fastBCR* identified a total of 13,971 HC based clonal families, with each family containing, on average, 10 unique amino acid sequences; cluster sizes ranged from 3 to 74 sequences (Fig 1B). Based on these data, we then evaluated the concordance of LC sequences within each family. Specifically, LC concordance was defined by the fraction of dominant V and J gene usage and the similarity of the CDR3 amino acid sequences (Fig 1C). Details of the scoring method are provided in the Methods section.

### Heavy-chain clustering reflects light-chain consistency but exhibits substantial variation

**Fig 2:**
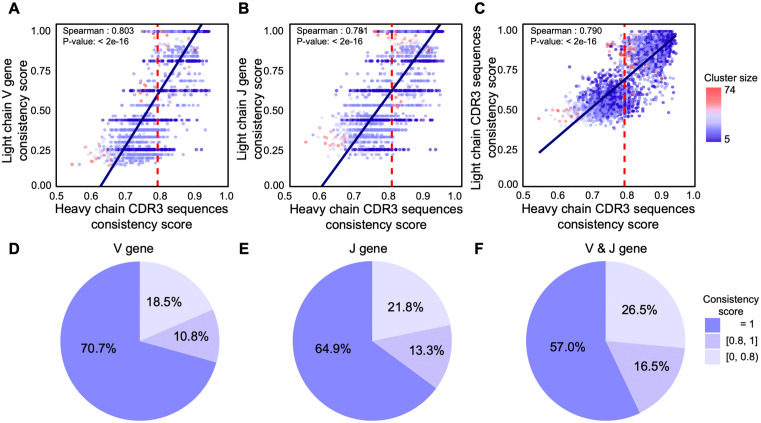
Evaluation of light chain consistency based on heavy chain clustering. (A–C) Scatter plots illustrating the correlation between LC V-gene/J-gene/CDR3 consistency scores and HC clustering consistency scores; point color indicates cluster size. (D–F) Pie charts showing the distribution of LC consistency scores in clusters with HC consistency score ≥ 0.8, classified by V gene, J gene, or the combination of both. (Clusters with size ≥5).

To ensure comparability across clusters in terms of V and J gene usage, we focused on clonal families containing at least five distinct HC amino acid sequences (2,686 clusters in total) for subsequent HC and LC concordance analyses. Across this large-scale dataset, we observed strong positive correlations between the consistency scores for LC V gene usage, J gene usage, and overall sequence identity, and those for HC concordance (Spearman’s ρ ~ 0.8, Fig 2A–2C). These findings suggest that HC-based clustering can, to a considerable extent, reflect LC consistency.

Nonetheless, even among clusters with high algorithmic confidence (HC sequence consistency score > 0.8), we uncovered notable variability in LC consistency. Specifically, 70.7% of these high-confidence clusters achieved a perfect LC V gene consistency score of 1, and 64.9% achieved a perfect LC J gene consistency score (Fig 2D and 2E). However, when considering both V and J genes together, only 57% of the clusters showed complete concordance for both V and J genes, suggesting a lack of complete consistency in the pairing of LC V and J genes (Fig 2F). Additionally, 26.5% of these clusters exhibited a dominant LC V-J gene usage score of less than 0.8, indicating that LC variability persists even in high-confidence clusters (Fig 2F).

Although slight discrepancies in the calculated metrics were observed due to the limited sequence count, similar trends were consistently found in clusters with fewer than five members, reinforcing the robustness of our observations (S1 and S2 Figs). These findings highlight that, while HC-based clustering partially captures key features of paired strands, the substantial variability in LC consistency introduces potential confounding factors, leading to artifacts (chain-mixed clusters) in B cell clonal family inference.

### Systemic biases in heavy-chain-only clustering reveal the limits of partial immune information

**Fig 3:**
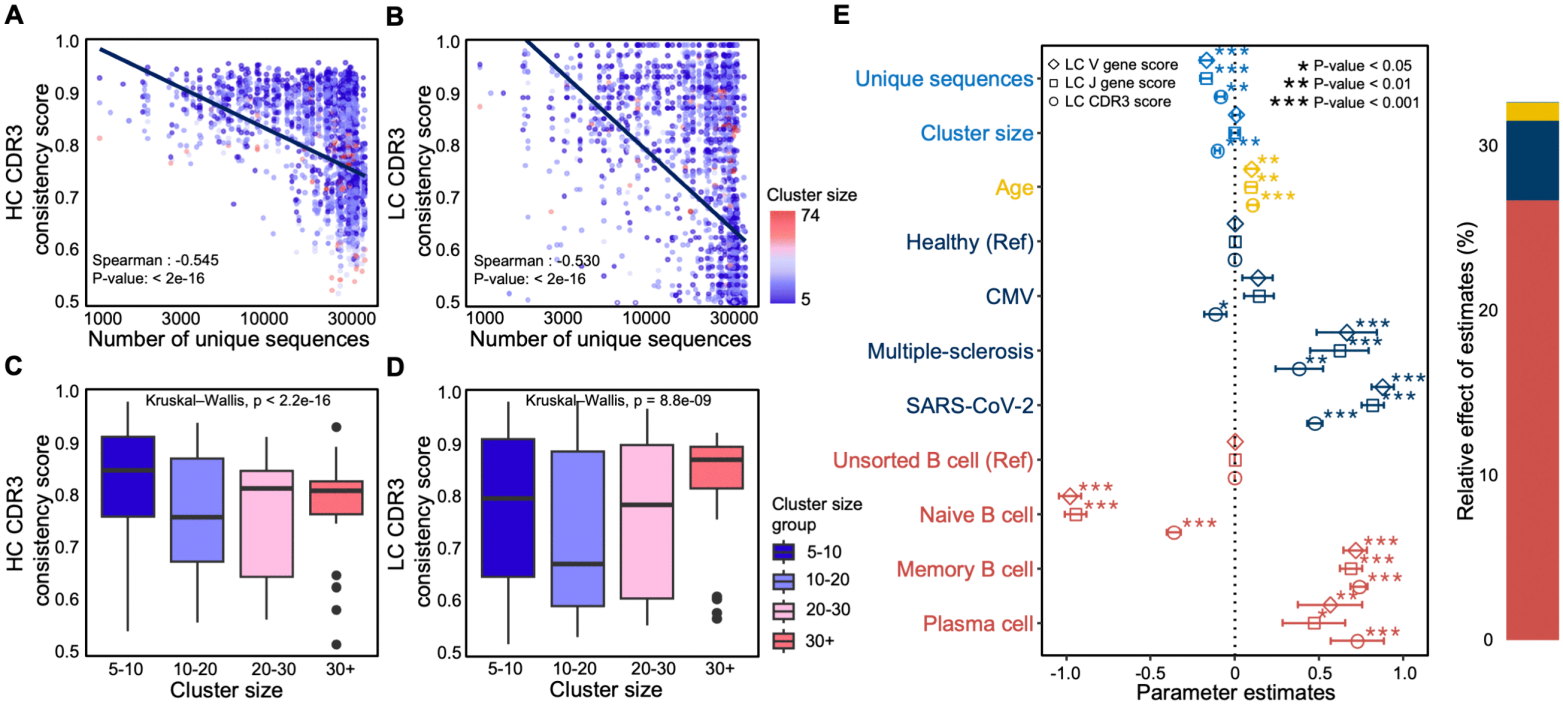
Multivariate regression analysis identifies potential factors driving chain-mixed clusters. (A–B) Scatter plots showing the correlation of sequencing depth and LC/HC consistency; point color indicates cluster size, and lines represent fitted regression trends. (C–D) Box plots of LC and HC consistency stratified by cluster-size groups. The black horizontal line denotes the median, boxes span the interquartile range, and dots represent outliers. Multivariate regression analysis. The left section presents regression coefficients and confidence intervals for each variable, where circles, diamonds, and squares indicate separate models using LC consistency, V-gene consistency, or J-gene consistency as the dependent variable. The bar chart on the right displays each variable’s contribution to explained variance. Asterisks (*, **, ***) denote p < 0.05, p < 0.01, and p < 0.001, respectively.

To elucidate the factors that influence LC consistency, we conducted a systematic investigation of multiple variables that could impact the inference results. We first considered sequencing depth, which directly dictates how comprehensively the BCR repertoire is captured; it may therefore influence the formation and size of chain-mixed clusters. By examining the relationship between sequencing depth and both HC and LC consistency, we observed a significant negative correlation (Spearman’s ρ of -0.545 and -0.53, respectively; Figs 3A, 3B, and S3), suggesting that higher sequencing depth captures a broader spectrum of B cell clones, increasing intra-cluster diversity and, consequently, the likelihood of chain mixing.

Although deeper sequencing can improve the overall capture of BCR clones, its effect on cluster size and LC consistency is variable. Analysis of the distribution of HC and LC consistency scores across different cluster sizes revealed that medium-sized clusters (5–30 sequences) had significantly lower scores than larger clusters (more than 30 sequences; Figs 3C, 3D, and S3). Thus, while greater sequencing depth enables more comprehensive BCR repertoire coverage, medium-sized clusters appear more prone to chain mixing. Conversely, larger clusters tend to achieve higher LC consistency, presumably due to the more robust representation of true BCR clonotypes in these highly expanded cells.

Building on this depth-dependent increase in intra-cluster diversity and mixing, we next asked whether sample composition further modulates LC consistency. Because most bulk repertoires do not strictly separate cell types, we incorporated naive B cell data as an unactivated control to distinguish background SHM and incidental small clones from activation- or disease-driven expansions. Accordingly, beyond sequencing depth and cluster size, we included donor age, disease category, and B-cell subtype as covariates. To quantify the relative contributions of these factors and identify key predictors, we employed a multivariate linear regression model [41] (see Methods). Disease types and cell types were encoded as categorical variables (dummy variables) using healthy samples and unsorted B cells as baselines, respectively. Consistent with the univariate analysis, sequencing depth showed significant negative correlations with LC consistency, whereas the effect of cluster size was not intuitive (Fig 3E). Donor age exhibited a positive correlation, potentially reflecting higher numbers of memory B cells and lower frequencies of naive cells in older individuals. Disease status also had a strong effect: compared with healthy controls, certain diseases such as multiple sclerosis and SARS-CoV-2 infection had positive regression coefficients exceeding 0.5, suggesting enhanced clonal expansion and affinity maturation in these contexts. In contrast, CMV-infected samples did not display a significant association with these patterns, likely due to the virus’s long-term coexistence with the host immune system, which may dampen B cell clonal expansion signals. Notably, B cell subtype emerged as the dominant predictor, explaining more than 25% of the variation in LC consistency. In particular, naive B cells showed strongly negative regression coefficients (less than -0.5), whereas memory and plasma cells were positively associated (greater than 0.5), underscoring that antigen-driven expansion and affinity maturation are key contributors to the sequence convergence observed within B cell clonotypes.

### Publicness and pre-structured immune motifs in naive clonotypes

**Fig 4:**
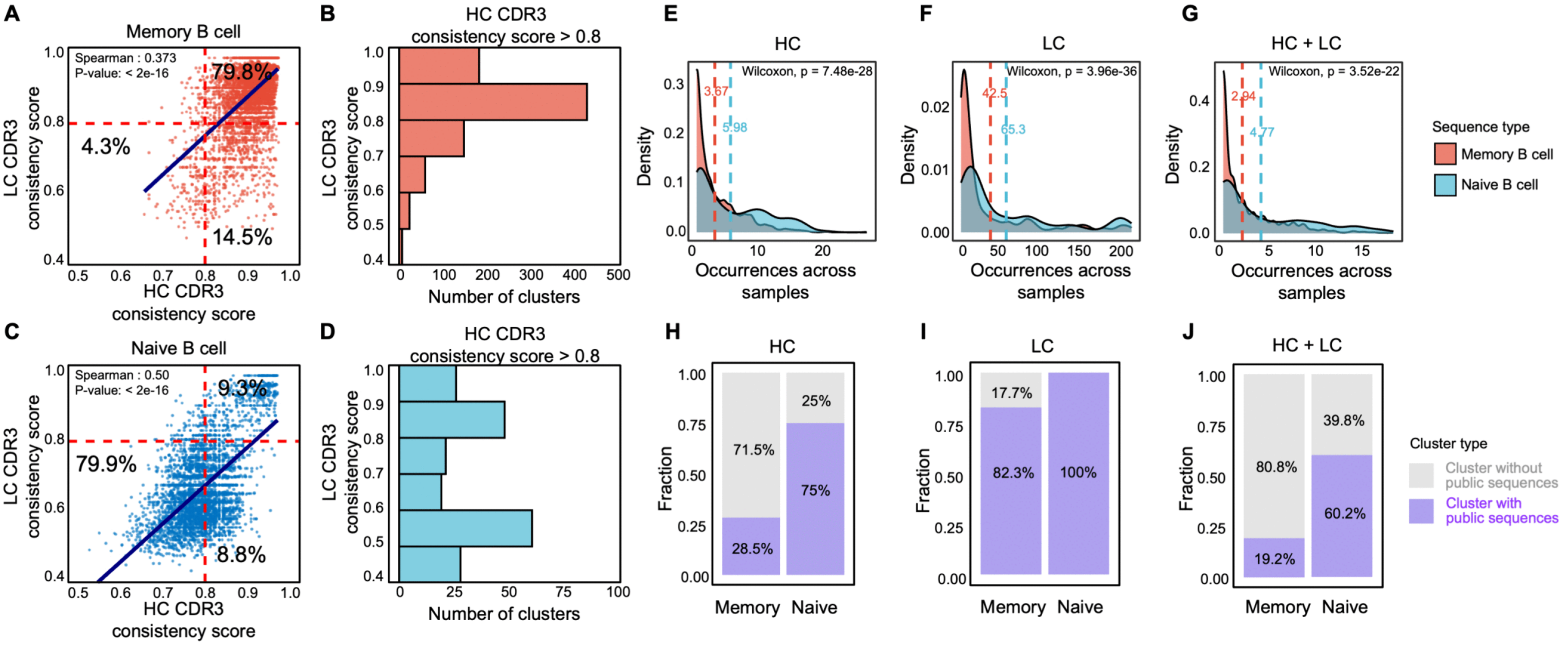
Comparisons of LC and HC consistency in naive and memory B cells. (A, C) Scatter plots showing the correlation of LC and HC consistency scores of clonal clusters in memory and naive B cell samples. (B, D) Histograms showing the LC consistency distributions for sequences with HC consistency ≥0.8 in the two cell types. (E–G) Density plots illustrating how frequently the sequences occur across all samples, with dashed vertical lines marking the mean frequency of sequence occurrence in different samples. (H–J) Bar plots depicting the proportion of clonal clusters potentially containing public antibodies, distinguished by color for clusters that do or do not contain public sequences. Panels (E–J) focus on sequences belonging to clusters in the upper-right quadrant of (A, C) (i.e., cluster size ≥5 and LC/HC consistency ≥0.8).

Our regression analysis indicated that the presence of naive B cells, either in pure naive or in unsorted repertoires, had a significantly negative effect on LC consistency. This effect can be explained by biases in V(D)J recombination at the HC locus, which preferentially generate naive B cells with highly similar or even identical HCs. Because such similar HCs can be paired with distinct LCs, HC–based clustering tends to group B cells that share the same HC but may differ in their LCs into the same clonal cluster, thereby giving rise to chain-mixed clusters. To further investigate this, we compared HC and LC consistency between naive and memory B cells. As shown by scatter plots of HC vs. LC sequence consistency scores, memory B cells predominantly occupied high-consistency regions (score > 0.8), accounting for 79.8% of their clusters (Fig 4A and 4B), consistent with the expected sequence homogeneity within antigen-experienced clonal clusters. By contrast, naive B cell clusters were strongly enriched in regions of reduced or imbalanced consistency (overall comprising 79.9% of naive clusters; Fig 4C and 4D). Notably, in both memory and naive cells, a subset (14.5% and 8.8%, respectively) of clusters still exhibited high HC consistency but low LC consistency, and these clusters represent the primary source of chain-mixed clusters.

Despite the prevalence of chain-mixed clusters, a minority of clusters (9.3%) in naive B cells exhibited high HC and LC consistency (Fig 4C). These clusters, though less frequent, warrant deeper investigation into their formation mechanisms, as they represent clonal clusters with highly homogeneous HCs and LCs within a compartment that has not yet undergone overt antigen-driven selection. To explore this, we assessed the degree of inter-sample sharing for sequences within these high-consistency clusters. Sequences from naive B cell in such clusters showed significantly higher inter-sample sharing than those from memory B cells, and this pattern was consistent across HC, LC, and paired-chain analyses (Fig 4E-4G). At the cluster level, 75% of high-consistency naive B cell clusters contained HC sequences shared by at least 10 samples, significantly higher than the 28.5% observed in memory B cells (Fig 4H). A similar pattern was observed for LC sharing (Fig 4I). While the overall proportion of shared sequences decreased when combining both HC and LC data, high-consistency clusters in naive cells remained significantly more shared compared to memory B cells (Fig 4J). Moreover, high-consistency clusters in naive B cells exhibited significantly lower levels of SHM compared with truly clonal clusters in memory B cells (S4 Fig). Together, these findings suggest that the HC-LC high-consistency clusters in naive B cells, which we defined as pseudo-clonal clusters, are often composed of highly public sequences that may be preferentially generated during B cell development rather than through antigen-driven selection.

### Construction of a public BCR sequence prediction model for identification of potential pseudo-clonal clusters

**Fig 5:**
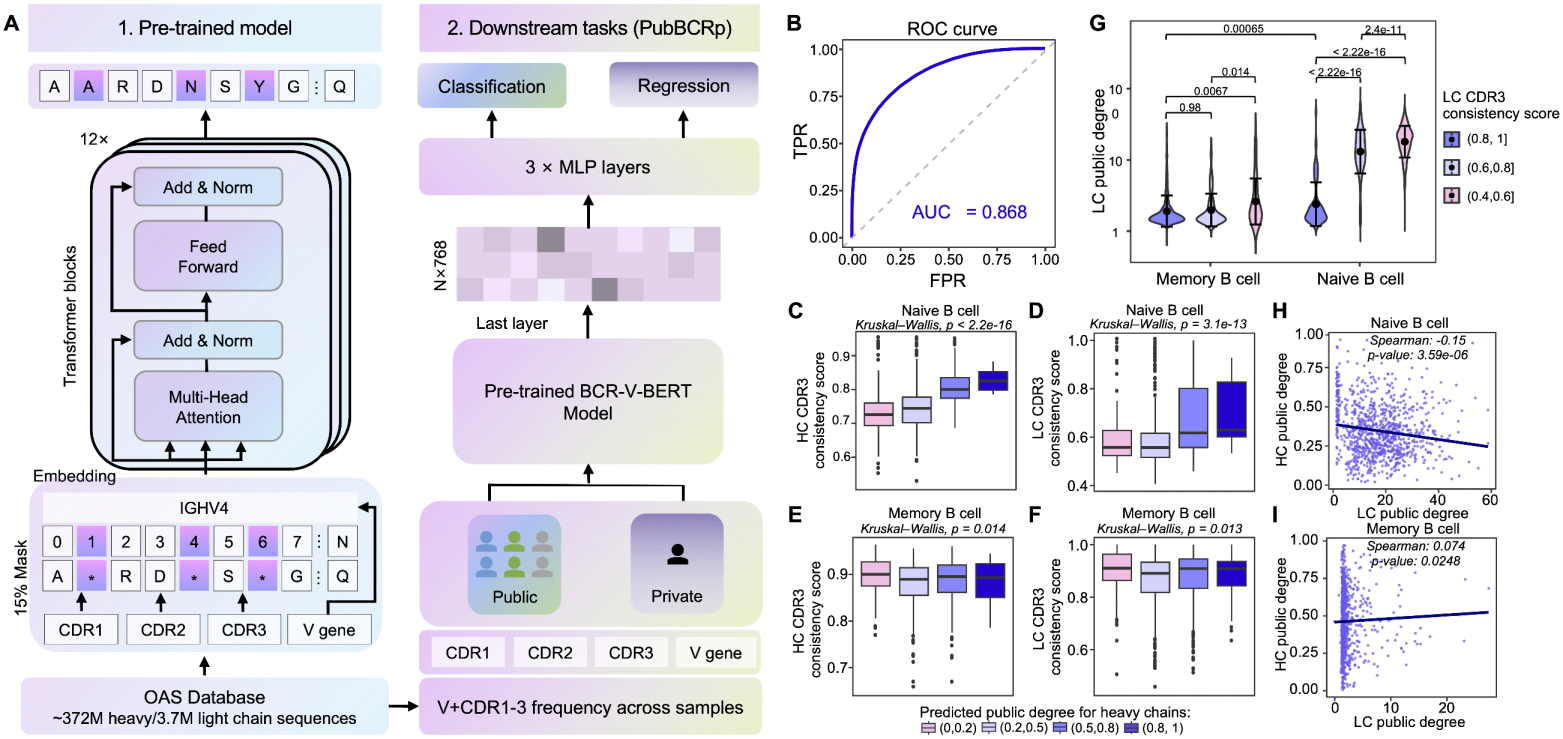
Construction of public BCR prediction model to predict high-concordance clusters. (A) Model architecture. Left (pretrained model): Inputs are LC and HC sequences, including CDR1–3 amino acid sequences and V-gene identities. A multi-layer Transformer (BCR-V-BERT) extracts sequence features via attention. Right (downstream tasks): (a) Heavy-Chain Classification: Sequences with occurrence frequency >10 are designated as public antibodies (positive), matched against frequency-1 sequences (negative) with the same V-gene distribution. Input features are HC embeddings; outputs are binary labels indicating public vs. non-public sequences. (b) Light-Chain Regression: Uses LC embeddings as input; the occurrence frequency of each LC sequence (across multiple samples) is the regression label, representing publicness. (B) Heavy-chain classification model ROC curves, illustrating performance on the validation dataset; AUC represents area under the curve.(C–F) Plots examining how heavy-chain publicness score groups relate to LC and HC consistency in naive (C, D) vs. memory (E, F) cells. The publicness score is the mean predicted probability of sequences in a cluster being public (range: 0–1). Four bins are defined by the publicness score (1, [0.5, 0.8), [0.2, 0.5), [0, 0.2)), color-coded from dark to light. Black lines mark medians; outliers appear as points. (G) Violin plots showing differences in heavy- and light-chain public scores for memory and naive B cells, stratified by LC consistency groups. The black dots indicate mean values, vertical lines represent standard deviations, and colors denote distinct LC groups. (H–I) Scatter plots depicting the correlation between HC and LC publicness scores in memory cell (H) and naive cell (I). Lines represent fitted regression trends.

While chain-mixed clusters can be readily corrected when paired data are available, pseudo-clonal clusters driven by public sequences remain difficult to diagnose from clustering alone. We therefore sougnt a scalable way to quantify the publicness of BCR sequences and clusters, so that candidate pseudo-clonal structures could be systematically characterized and flagged fo downstram handling. We hypothesized that these sequences possess distinct characteristics that can be learned and predicted using machine learning. To enhance prediction performance, we first conducted self-supervised pretraining on a large-scale BCR sequence dataset, which allowed for improved feature representation. The pretraining model, illustrated in Fig 5A, employs a classical BERT [42] (Bidirectional Encoder Representations from Transformers) framework consisting of 12 stacked Transformer encoder layers, each containing 12 multi-head attention modules and a hidden layer dimension of 768. The model takes as input the V gene and CDR1–3 amino acid sequences of the HC or LC, with random masking employed during training to capture contextual information through bidirectional attention. By training on 372 million HC and 3.7 million LC sequences from OAS unpaired BCR repertoires, the model learned a rich, high-dimensional representation of BCR repertoires (S5 Fig, see Methods for details).

For public sequence prediction, we used the final layer of the pretrained model to obtain embedded representations of BCR sequences, which were then input into a three-layer fully connected network for classification and regression tasks. For the HC model, we classified high-frequency (more than 10 occurrences) and low-frequency (single occurrence) sequences as positive and negative samples, respectively, and built a binary classification dataset by balancing the data distribution. In contrast, due to the high frequency of LC public sequences, a binary classification model was not suitable. Thus, we developed a regression model to predict the public BCR frequency for LCs. In this model, the frequency of LC sequence appearance across samples was used as the regression target. The final datasets were partitioned into training, testing, and independent validation sets in an 8:1:1 ratio.

The HC classification model demonstrated robust performance, achieving an AUC of 0.868, with precision and recall around 0.78 on the validation dataset (Figs 5B and S6). Under identical evaluation settings, the model consistently outperformed both the embedding-based antiBERTa [43] baseline and the probabilistic generative model OLGA [44,45] across multiple standard evaluation metrics, including Accuracy, Precision, Recall, F1 score, and AUROC (S7 Fig). After model establishment and validation, we calculated a public score for each clone cluster, which is the average of all sequence public prediction probabilities within the cluster, ranging from 0 to 1. We further grouped clonal clusters based on their public scores and analyzed the relationship between HC public scores and HC and LC consistency scores. In naive B cells, clusters with high HC public scores exhibited higher HC and LC consistency (Fig 5C and 5D), indicating that HC-LC high-concordance clusters in naive B cells are enriched for public sequences, consistent with the pseudo-clonal signature. In contrast, in memory B cells, cluster concordance remained high regardless of public score (Fig 5E and 5F), suggesting that public sequence enrichment contributes less to clonal structure in antigen-experienced repertoires. Plasma cells showed a similar pattern to memory B cells (S8 Fig). Notably, unsorted B cell samples displayed a public score dependent pattern resembling that observed in naive B cells (S8 Fig), likely due to the influence of naive-derived public sequence signals in mixed repertoires.

The LC regression model also demonstrated strong predictive capability on the validation dataset, with a Spearman correlation of 0.735 and a Root Mean Squared Error (RMSE) of 4.478 (S6 Fig). However, when we used the LC public score to compare the difference between clusters, the results differed from those of the HC model. Although naive B cells exhibited significantly higher LC public scores than memory B cells, these clusters in naive cells had lower LC public degrees compared to their own low-consistency regions (Fig 5G). Further analysis revealed a negative correlation between HC and LC public degree within naive B cell clusters (Fig 5H, Spearman’s ρ = -0.294, p < 0.01) but not in memory B cell (Fig 5I, Spearman’s ρ = 0.074, p = 0.0248). These findings suggest that the maturation process in naive B cells likely involves selective mechanisms that shape HC-LC pairing in a non-random manner. If pairing were completely random, highly public HCs would be expected to preferentially pair with highly public LCs, which is not observed.

### Integrating paired-chain logic and publicness prediction to improve clonal family inference

**Fig 6:**
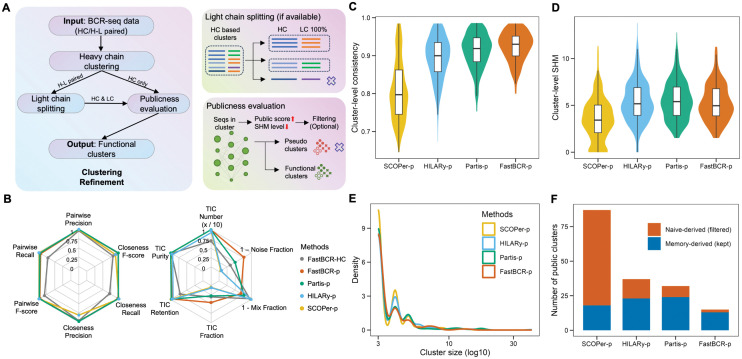
Improving BCR clustering via light-chain splitting and public sequence aware refinement. (A) Schematic illustrating the clustering optimization pipeline, which incorporates LC V/J gene splitting and publicness evaluation. (B) Radar plots comparing original and improved methods with multiple paired-chain clonal family inference approaches across normalized clustering metrics. (C–D) Violin plots comparing cluster level consistency scores (C) and somatic hypermutation (SHM) load (D) across real unsorted BCR repertoire datasets using different paired-chain clustering methods. (E) Distribution of cluster sizes across different clustering methods. (F) Bar plot showing the number of clusters identified as public by each method, with colors indicating the cellular origin of clusters based on SHM load.

The above analysis of large-scale BCR data reveals biases in clonal family inference when relying solely on HC sequences. To enhance the accuracy and representativeness of B cell clonal inference, we developed an optimized clustering strategy, *fastBCR-p*, that incorporates LC V-J gene splitting and public sequence aware refinement. First, when paired data available, we applied a splitting strategy based on LC V-J genes to sub-cluster HC based clusters, thereby reducing chain-mixing artifacts arising from LC diversity. In addition, we introduced an evaluation step to identify clusters enriched for highly public BCR sequences, as elevated publicness may arise from naive like convergent generation rather than true clonal expansion (Fig 6A). To this end, a cluster-level publicness score was defined as the arithmetic mean of the publicness probabilities of all HC/HC–LC sequences within each cluster. Putative public clusters were identified using a data-driven cutoff corresponding to the upper tail (top 10%) of the publicness score distribution (S9 Fig). Considering the potential activation of public clones, SHM load was quantified as the mean SHM across HC/HC–LC sequences to assess the cellular origin of public clusters. An SHM-based cutoff was subsequently applied to classify public clusters as naive-derived or memory-derived, by ROC analysis using annotated naive and memory B-cell clusters, with the threshold corresponding to the maximal Youden index (S9 Fig).

To rigorously assess the performance of our improved method, we conducted comprehensive evaluations on both simulated and real-world datasets and compared it against existing paired-chain clonal family inference methods, including SCOPer [28], Partis [46], and HILARy [47]. In the simulated dataset, designed with clear “gold standard” annotations (spanning 3,447 sequences from 10 clonal families plus 3,504 noise sequences; see Methods), we compared the HC-based clustering method, our improved approach, and additional baseline methods under the same evaluation framework. We assessed the clustering performance using multiple complementary metrics that capture distinct aspects of clustering quality. Purity measures the accuracy of a cluster in assigning true positives, while Precision quantifies the proportion of true positive assignments relative to all assignments made to a given cluster. Recall measures the proportion of true positive assignments relative to all true positives, reflecting the ability of a method to recover complete clone-related groups. In addition, the F1 score, defined as the harmonic mean of Precision and Recall, provides a balanced assessment of clustering performance. The true-inferred clusters (TIC) fraction highlights the proportion of relevant clusters retained, reflecting the extent to which the method preserves meaningful information (see Methods). Performance differences on the simulated data are visualized using radar plots, with TIC retention is normalized to (0, 1) to ensure a consistent scale across metrics (Fig 6B). Collectively, the results show that the improved method consistently outperforms the original HC-based method and achieves comparable performance compared with other paired-chain methods across most evaluated metrics, indicating that our improved strategy effectively refines clustering performance.

Moreover, we evaluated the performance of different methods using real-world unsorted BCR dataset. The results show that our improved method achieves varying degrees of improvement in both cluster consistency score and cluster-level SHM compared with existing methods (Fig 6C and 6D), indicating higher accuracy in grouping clonally related BCR sequences and better preservation of biologically meaningful SHM patterns. Notably, the cluster sizes obtained by different methods exhibit similar distributions (Fig 6E), suggesting that the observed performance gains are not driven by differences in clustering granularity. In addition, we quantified the number of public clusters and potentially filtered clusters using the public sequence evaluation pipeline. The results show that all methods are influenced by the presence of public sequences to a varying degree and retain a subset of naive-like pseudo-clonal clusters (Fig 6F). Overall, these findings highlight the value of incorporating dedicated filtering and identification steps for public sequences, which facilitate the discrimination between truly activated clusters and spurious pseudo-clonal clusters.

## Discussion

Decoding the specificity and diversity of BCR repertoire are critical for understanding B cell immune functions and antibody generation. In this study, we systematically evaluated the performance and limitations of BCR clonal family inference based solely on HC sequences. Through in-depth analysis of large-scale paired-chain data, we found that while clustering based on HCs alone can reflect certain aspects of clonotype characteristics, it may subject to two major sources of limitations that give rise to chain-mixed or pseudo-clonal clusters and thereby complicate the identification of true clonal populations in B cell repertoire data: (i) chain-mixed clusters resulting from similar HCs being paired with different LCs, and (ii) highly homogeneous naive clusters (pseudo-clonal clusters), a limitation that is most pronounced in datasets containing substantial naive cell fractions, particularly unsorted samples. It is worth noting that these two phenomena are not entirely independent, but are both likely driven by high BCR generation probabilities, particularly for the HCs. To overcome these limitations, we propose a method that incorporates strategic LC segregation and public sequence aware refinement., significantly improving the accuracy and completeness of clonotype reconstruction. This advancement not only provides more precise tools for studying B cell immune functions but also offers new insights for antibody optimization.

Our findings suggest that similar HCs paired with different LCs is a major contributor to the formation of chain-mixed clusters, a phenomenon influenced by multiple factors. High sequencing depth, while enhancing the capture of BCR sequence information, also introduces more LC mixing. We also observed that samples from different disease types and age groups exhibited varying LC consistency, indicating that HC clustering results are not only influenced by data volume but also by the biological characteristics of the samples. As sample age increases, LC consistency tends to improve, which aligns with the findings that expansion of memory B cells and the decrease in naive cells in older individuals [48]. Furthermore, disease samples showed a significant increase in LC consistency compared to healthy samples, suggesting that B cells in diseased states undergo clonal expansion and affinity maturation following antigenic stimulation. For example, SARS-CoV-2 infection resulted in relatively high and concentrated LC consistency scores, while CMV infection did not show significant associations with the same clonal expansion and maturation patterns. This is likely due to CMV’s ability to persist in a latent state within the host for extended periods. Over time, the immune system adapts to this chronic infection, and the B cell response becomes more regulated and less responsive to antigenic stimulation. In such cases, the virus may induce a more stable immune environment with diminished B cell clonal expansion signals, as the immune system has already developed long-term memory responses that help maintain immune tolerance to the virus. This dampening effect on B cell activation and clonal expansion may explain the lack of significant association with the observed patterns in CMV-infected samples. These findings underline the importance of considering the specific immune context in which B cell responses occur, as different diseases may drive distinct patterns of immune activation or suppression.

Naive B cells, which have not undergone selective antigenic stimulation, theoretically exhibit high sequence diversity and low clonotype expansion. However, our study reveals a significant presence of high consistency clonotypes in naive B cells. Further analysis showed that high consistency clonotypes in naive B cells are predominantly composed of public BCR sequences, whereas the proportion of public clusters in memory B cells is much lower. This supports our hypothesis that the presence of public BCRs is the main driver of high-consistency clonotypes in naive B cells, likely due to the preferential expression of germline heavy-chain genes and their higher generation probability. To validate this hypothesis, we used machine learning models to predict the clonotype characteristics of naive B cells, which further reinforced the critical contribution of germline public antibodies to the formation of high consistency clonotypes.

Moreover, we observed that the LC consistency in naive B cells is not simply a result of random pairing but may also be regulated by specific biological mechanisms. Our results show that although HCs in high-consistency naive B cell clonotypes exhibit high public BCR characteristics, the LCs within these clonotypes display lower public characteristics compared to other clonotypes. This suggests that the degree of publicness in HC and LC sequences is not solely determined by their recombination probability, and is likely influenced by antigen-driven convergent selection processes. [49,50] The negative correlation between the publicness of HC and LC sequences further supports the idea that the pairing process for these two chains may be influenced by different factors, rather than relying on independent generation mechanisms. We speculate that during B cell development, the HC and LC combination preferences may be linked to physiological requirements such as antibody diversity and immune tolerance. This inconsistency in publicness may contribute to maintaining the diversity and flexibility of the B cell repertoire, preventing the immune response from being restricted to a single pattern.

Building on these findings, we propose an improved strategy based on LC splitting and public BCR filtering, demonstrating significant advantages in processing large-scale BCR data. Recent studies [51] have evaluated eight different BCR heavy-chain clonal family inference methods, ranging from traditional sequence alignment to more complex probabilistic models and natural language processing techniques. While these methods differ in terms of the number and distribution of reconstructed clonotypes, the results suggest that probabilistic models did not significantly outperform simpler sequence alignment methods in terms of accuracy. In fact, many methods, despite achieving high HC consistency, have not effectively addressed the LC consistency issue, underscoring the limitations in handling paired chains. Our approach, by introducing LC splitting and public antibody filtering, can better identify and process “pseudo-clonotype clusters” caused by public antibody sequences, significantly improving the accuracy of inference. Especially in cases involving naive B cells and mixed samples, traditional HC clustering methods are often influenced by LC mixing effects, leading to inaccurate inferences. In contrast, our method enables efficient and accurate identification of clonotype diversity and paired-chain consistency, providing a more reliable tool for BCR analysis.

Although our improved strategy has shown significant effectiveness in both simulated and real data, several areas remain for further exploration and optimization: 1) Handling and optimizing large-scale datasets: with the development of single-cell or paired-chain sequencing technologies, we will face increasingly large paired-chain datasets. Our method needs further optimization to balance efficiency and accuracy to ensure scalability in large datasets. 2) Exploring the biological functions of public antibodies: the public antibody prediction model developed in this study mainly serves to filter interference and improve clustering accuracy. However, the biological function and evolutionary significance of public antibodies remain underexplored. Future studies should further investigate the role of these antibodies in immune responses and how they contribute to immune system homeostasis. This could provide deeper insights into B cell evolution, antibody drug development, and personalized vaccine design.

## Materials and methods

### Heavy-chain based clonal family inference

We employed *fastBCR* [39,40] to infer HC clonal families, with inference performed independently within each sample. *fastBCR* uses a *k*-mer–based method centered on germline-derived seed indices, followed by a rapid pre-clustering step and an optimized clustering step:

a. Pre-Clustering: Sequences were partitioned by V/J gene types and sequence length (VJ groups). Within each VJ group, *fastBCR* extracted six *5*-mer substrings from each junction amino acid sequence as “seeds,” aggregating sequences containing identical 5-mers into preliminary seed clusters. Next, seed clusters with similar lengths and positions were merged, accounting for potential Indels.
b. Optimized Clustering: A dynamic programming approach then integrated multiple seed-cluster results to yield candidate clonal families. A consensus score was calculated for each family, filtering out low-quality clusters. The result was a set of stable, representative clonal families.

In this study, *fastBCR* was run with the following parameters:

- cluster_thre = 3: default threshold for seed merging.
- overlap_thre = 0.1: minimal overlap ratio required for cluster merging.
- consensus_thre = NA: no filtering on consensus scores was applied, allowing for a comprehensive evaluation of both heavy- and light-chain correlations.

### Evaluation of light chain consistency

To evaluate LC consistency, we defined a V/J gene consistency score and intra-cluster junction sequence consensus score. The V/J gene consistency score quantifies the degree of the dominate V gene and J gene usage within a given clonal family. It is calculated as:


$$V\;or\;J\;gene\;consistency\;score=\frac{\;n_{\text{max}}\;}{N}$$

Here, N represents the total number of sequences in the cluster, and $n_{\text{max}}$ is the count of sequences with the highest frequency V/J gene. This score ranges from 0 to 1, where 1 indicates complete concordance in V/J gene usage across all sequences in the cluster.

Additionally, the intra-cluster junction (CDR3) sequence consistency score measures the similarity of junction sequences across sequences within the same clonal family. To calculate this score, we first perform a multiple sequence alignment (MSA) on the junction sequences of the members within a specific clonal cluster. This alignment helps identify the most frequently occurring amino acids at each position. The resulting sequence, known as the consensus sequence, represents the predominant amino acid composition shared by the members of the clonal family. At each position, positional consensus score is defined as the consistency of the other member sequences in the cluster with the consensus sequence. Then the consensus score is determined by calculating the average of the positional consensus score.


$$CDR\text{3}\;seuquence\;consistency\;score=\frac{\sum_{i}^{l}positional\;consensus\;score}{l}$$

The consistency score ranges from 0 to 1, where a score closer to 1 indicates high homogeneity in the junction sequences.

The combination of these two metrics allows for a comprehensive evaluation of LC consistency, providing insight into the stability of both gene usage and junction sequence diversity within clonal families.

### Multivariate regression analysis

To investigate how sequence features, sample attributes, and disease conditions affect clonal family consistency, we employed generalized linear mixed models (GLMMs) via the glmmTMB [41] package in R. For each continuous response variable (V score, J score, and sequence consensus score), a Beta regression model with a logit link was constructed. Fixed effects included donor age, total unique sequences per sample, cluster size, B cell subtype (using unsorted cells as the reference level), and disease status (using Healthy as reference). The response variables were assumed to follow a Beta distribution over the (0, 1) interval; extreme 0 or 1 values were replaced with a small offset (e.g., ${\text{1}\text{0}}^{-\text{6}}$ or ${\text{1}-\text{1}\text{0}}^{-\text{6}}$) to satisfy model assumptions.

We computed marginal $R^{\text{2}}$($R_{m}^{\text{2}}$) and conditional${\;R}^{\text{2}}$ ($R_{c}^{\text{2}}$) via the r.squaredGLMM() function from the MuMIn package to evaluate the explanatory power of the fixed and random effects, respectively. Since partial $R^{\text{2}}$ for each predictor is not directly available in GLMMs, we used standardized coefficients via the standardize_parameters() function in the parameters package to compare their relative importance on a unified scale. Final models were presented with coefficients, standard errors, p-values, and $R^{\text{2}}$ indices, alongside visualization (e.g., forest plots) to illustrate model outcomes and explanatory strength.

### Public BCR sequence prediction model

#### Pretraining data collection and processing.

To train the self-supervised antibody sequence model, we curated a large-scale single light/heavy chain dataset from the OAS database. The raw data underwent systematic cleaning and preprocessing to ensure quality and consistency. We discarded all sequences containing non-standard amino acid characters or whitespace, retaining only the 20 canonical amino acids. Functional annotations were obtained from the original output file, extracting the CDR region that define the antibody binding interface (CDRH1, CDRH2, CDRH3 for heavy chains, and CDRL1, CDRL2, CDRL3 for light chains). To simplify V-gene complexity, we retained only the major allele, removing allele-specific designations (e.g., IGHV3–23*04 truncated to IGHV3–23). We then deduplicated the dataset based on CDR1–3 sequences and V-gene annotations, yielding 372,028,240 unique HC sequences and 3,705,441 unique LC sequences.

#### Pretraining model architecture and training.

We introduced BCR-V-BERT, an adaptation of the classic Bidirectional Encoder Representations from Transformers (BERT [42]). BCR-V-BERT comprises a 12-layer stacked Transformer encoder with a hidden dimension of 768 and 12 multi-head self-attention modules per layer. A bidirectional attention mechanism captures contextual dependencies in BCR sequences. Additionally, our model features two embedding layers: (1) an amino acid embedding layer for 20 canonical amino acids and 5 special tokens (PAD “$”, MASK “.”, UNK “?”, SEP “|”, CLS “*”) and (2) a V-gene embedding layer capturing gene-level features via a dedicated V-gene vocabulary.

We employed a masked language model (MLM) objective, randomly masking 15% of the amino acid tokens. Among these, 80% were replaced by the “MASK” token, 10% by a random amino acid, and 10% were left unchanged. We randomly selected 10,000 BCR sequences as a test set and used the remainder for training. We employed a training setup with a batch size of 64 and a learning rate of 3e-5, optimizing for 20 epochs on three NVIDIA 3090 GPUs. The training took approximately 36 hours to converge. After training, the model’s final output layer served as feature embeddings for downstream applications. We trained two versions of the model: BCR-V-BERT-h for HCs and BCR-V-BERT-l for LCs, with the latter downsampling sequences from highly abundant V genes to balance the dataset.

#### Construction of public BCR prediction models.

To address the challenge of predicting public BCRs, we developed two separate models: a regression model for predicting the publicness of LCs and a binary classification model for HCs.

To construct the public HC dataset, we labeled HC sequences with ≥10 occurrences under the same V-gene and CDR pattern as positive samples (370,056 total) and sequences occurring exactly once as negative samples (353,315,928 total). To mitigate distributional bias, we performed 1:1 matching of positive and negative examples based on V-gene identity, resulting in 370,056 negative examples and a final dataset of 740,112 HC entries. This dataset was split into training, validation, and test sets in an 8:1:1 ratio.

Using the pretrained BCR-V-BERT model, we embedded the HC CDR1–3 sequences and V-gene information into feature vectors. A three-layer fully connected neural network was employed to distinguish public antibodies from private (non-public) ones. Binary cross-entropy loss was used as the loss function to optimize the model, and the Adam optimizer was applied with a learning rate of 1e-3 and weight decay of 1e-5. Training was conducted for 50 epochs with a batch size of 1280, utilizing three NVIDIA 3090 GPUs.

Model evaluation was performed using the following metrics: Area Under the Curve (AUC): Used to assess the model’s ability to distinguish between public and private antibodies across various threshold settings. Precision, Recall, and F1-score: Precision measures the proportion of correctly identified public antibodies among predicted positives, recall evaluates the proportion of correctly identified public antibodies among actual positives, and F1-score provides a harmonic mean of precision and recall.

For the public LC dataset, sequences appearing ≥5 times with identical V-gene and CDR patterns were considered public (39,367 total). From the pool of sequences appearing <5 times (1,344,791 total), we performed 1:1 matching by V-gene identity across occurrence frequencies (4, 3, 2, or 1 time), resulting in a final dataset of 172,310 LC sequences. Appearance frequency served as the regression label. The dataset was split into training, validation, and test sets in an 8:1:1 ratio.

To construct the public LC regression model, we utilized the pretrained LC BCR-V-BERT model to embed the LC CDR sequences and V-gene information into feature representations. A three-layer fully connected network was then employed to predict the public score for LCs. The mean squared error (MSE) loss function was used to optimize the regression task, and the Adam optimizer was applied with a learning rate of 1e-3 and weight decay of 1e-5. Training was performed for 200 epochs with a batch size of 1280, using three NVIDIA 3090 GPUs. Spearman correlation coefficient was used to assess the alignment between predicted and true publicness scores, indicating the model’s ability to capture rank-order relationships.

### Implementation details of fastBCR-p

To improve the accuracy of capturing true clonal cluster features, fastBCR-p incorporates two refinement steps: LC–based splitting and public sequence aware refinement. Following initial HC-based clustering, clusters are further subdivided according to distinct LC V–J gene usage when paired data are available, thereby reducing chain-mixing artifacts arising from LC diversity. Publicness scores are assigned to individual sequences using trained public BCR prediction models. For HC-only datasets, publicness is evaluated using the HC model, whereas for HC–LC paired datasets, heavy and light chains from the same cell are scored separately using the corresponding HC and LC models. Cluster-level publicness is defined as the arithmetic mean of the publicness probabilities across all sequences within each cluster. To identify putative public clusters, heavy- and light-chain publicness scores are standardized using z-score normalization, and a data-driven cutoff corresponding to the upper 10% of the standardized score distribution is applied.

SHM load is subsequently used to assess the cellular origin of public clusters. Cluster-level SHM is defined as the mean SHM across all HC/HC–LC sequences within each cluster. As memory-derived B-cell clones are expected to exhibit higher SHM than naive-derived clones, an SHM-based cutoff is applied within public clusters to distinguish naïve-like pseudo-clonal clusters from bona fide memory-derived clonal expansions. Public clusters with low SHM are filtered out, whereas those with elevated SHM are retained. The SHM cutoff is determined by ROC analysis using annotated naïve and memory B-cell clusters, with the threshold corresponding to the maximal Youden index.

### Simulated data generation

To comprehensively evaluate the clustering performance, we designed a simulation procedure that closely reproduces the formation of BCR clonal families and the SHM patterns observed in vivo, while generating high-quality synthetic data sets for paired-chain analyses. The simulation begins with single-chain sequence modeling and proceeds to independently generate light and heavy chains, forming paired clonal families under the assumption that there is no intrinsic biological coupling between HC and LC rearrangements [52–55].

#### (1) Generation of ancestor sequences.

We first constructed representative ancestral sequences for each clonal family by randomly sampling from the human immunoglobulin gene repertoire. For HCs, V, D, and J gene fragments were randomly selected from IGHV, IGHD, and IGHJ, respectively, then assembled into a full-length HC DNA sequence. During V-D-J assembly, we introduced one to three random nucleotide insertions or deletions (Indels) at the D-J and V-D junctions to mimic natural junctional diversity. For LCs, V and J gene fragments were randomly selected from IGKV/IGKJ (kappa chain) or IGLV/IGLJ (lambda chain), with a 60% probability of generating a kappa chain and 40% a lambda chain. We introduced 0–2 Indels during V-J junction formation, resulting in slightly lower variability for LCs. Finally, we paired each newly generated HC ancestor with a randomly generated LC ancestor, designating them as the origin of a single clonal family.

#### (2) Simulation of somatic hypermutation.

To replicate multiple rounds of SHM within germinal centers, we defined a mutation rate μ ∈ (0,1), considering three mutational types (insertion, deletion, and substitution) at an 0.8:1:100 ratio to reflect observed biological preferences. Mutations were concentrated in and around CDR3 (±15 nucleotides upstream and downstream in heavy chains; ± 10 in light chains).

The simulation steps proceeded as follows:

a. Initial Amplification and Mutation: We began with an ancestral sequence and simulated five activated B cells undergoing clonal expansion. Each expanded sequence was then evaluated position by position for potential mutations, assigned according to the predefined type ratio.
b. Selection (Apoptosis): After each round of mutation, 40% of the newly generated variants were randomly discarded, modeling the selective apoptosis occurring within germinal centers.
c. Multiple Mutation Cycles: The amplification-mutation-selection steps were repeated for six rounds. Sequences surviving each round were carried forward to the next, culminating in a full clonal family that included both ancestral and mutated descendants.

Mutations included insertions, deletions, and base substitutions, with probabilities determined by a predefined model (e.g., a G base might be replaced by A at 70%, T at 15%, and C at 15%). Random number generators governed gene fragment selection, mutation locations, and mutation types, preserving randomness and diversity. LCs were subject to additional constraints relative to HCs, such as fewer Indels and smaller mutation windows, sometimes with a lower mutation rate, to mirror known biological differences.

#### (3) Data annotation and utility.

Simulated data were output in FASTA format with unique identifiers specifying chain type (IGH, IGK, or IGL), clonal family ID, and sequence number. Supplementary annotation files listed chain type, clonal family ID, V-gene end position (v_sequence_end), and J-gene start position (j_sequence_start), assisting downstream validation. These data served as benchmarks for evaluating methods designed to detect paired-chain clonal families, facilitating algorithm development and parameter tuning. After generation, we annotated and validated all sequences using IgBLAST v1.17.0 [56] with an IMGT germline database [57] snapshot downloaded on July 9, 2024. This procedure ensured conformity with canonical immunoglobulin structural features, accurate framework- and CDR-region definitions, and correct V(D)J gene assignments.

#### (4) Noise sequence generation.

Real-world BCR datasets often contain a nontrivial proportion of noise (singleton) sequences that do not belong to any true clonal family. To thoroughly evaluate our proposed paired-chain clustering approach, we introduced noise sequences that partially shared HC similarity—yet differed significantly at the LC level—and mixed them with the original clonal family data.

For noise HC sequences, we retained the same V/J gene annotations as in the original families (i.e., preserving the 5′ V-gene and 3′ J-gene regions), but introduced extensive random substitutions, insertions, or deletions in the junction (CDR3) region. Thus, while noise HCs overlapped in annotated V and J regions, they differed substantially in the junction. Consequently, algorithms relying solely on HC-based clustering could mistakenly incorporate these noise sequences into the original families.

By contrast, noise LCs were designed to deviate strongly from their counterparts in genuine clonal families. We randomly picked new V and J gene fragments (either kappa or lambda), introduced some Indels or truncations in the V-J junction, and applied higher-frequency point mutations or limited Indels across V or J regions, thereby considerably reducing sequence similarity with authentic light chains.

Collectively, this strategy yields noise sequences that retain partial HC overlap (V and J) while significantly diverging in CDR3 and/or LC sequences. HC-only methods may misclassify such noise, but inclusion of LC data helps distinguish inconsistent pairing, thereby improving overall clustering performance and illustrating the advantages of paired-chain approaches.

### Clustering evaluation metrics

Adjusted Rand Index (ARI): Evaluates agreement between clustering results and true labels (i.e., known clonal family assignments in simulated data), correcting for random chance. Let N be the total number of sequences; define $T_{\text{1}},T_{\text{2}},\ldots ,T_{r}$ as true label sets and $C_{\text{1}},C_{\text{2}},\ldots ,C_{s}$ as the clustering results. Let $n_{ij}=\left|C_{i}\cap T_{j}\right|$ be the number of samples in the intersection of $C_{i}$ and $T_{j}$, $a_{i}=\sum_{j}n_{ij}$ be the total samples in $C_{i}$, and $b_{j}=\sum_{i}n_{ij}\;$ be those in $T_{j}$. The ARI is calculated as:


ARI=∑ij(nij2)−∑i(ai2)∑j(bj2)(N2)12(∑i(ai2)+∑j(bj2))−∑i(ai2)∑j(bj2)(N2)

ARI values near 1 indicate near-perfect alignment with true labels, while values near 0 reflect random clustering.

The pairwise measure is specifically designed for the binary clustering task and focuses on the pairwise relationships between individual sequences. In the context of pairwise performance measures, a pair of sequences is considered true positive (TP) if the sequences are correctly clustered together in the inferred clusters. Conversely, the pair is counted as false positive (FP) if they are clustered together in the inferred cluster but separated in the original simulated cluster, and as false negative (FN) if they are clustered together in the original simulated cluster but separated in the inferred cluster. While pairwise measure provides valuable insights into pairwise similarities, it falls short in evaluating the clonal composition and the overall repertoire structure.

On the other hand, the closeness measure takes into account both clonal compositions and repertoire structure, but its computation necessitates the identification of the optimal correspondence between inferred and original clusters. In this case, the best correspondence between inferred clusters and simulated cluster should be determined first. For brevity, we denoted the simulated cluster as ${SC}_{i}$ (i = 1…N) and inferred clusters as ${IC}_{j}$ (j = 1…M). Since the clonal relationships were known in the simulation data, sequences in ${SC}_{i}$ can be assigned with the corresponding true label $L_{SCi}$. Furthermore, we assumed that ${IC}_{j}$ can be labeled by its most frequent label $L_{ICj}$. For each ${IC}_{j}$, the best correspondence refers to $\;{SC}_{i}$ having the same label with it (i.e., $L_{SCi}==L_{ICj}$). So far, three disjoint sets can be computed as $TP_{j}=\left|{IC}_{j}\cap {SC}_{i\left(L_{SCi}==L_{ICj}\right)}\right|$, $FP_{j}=\left|{IC}_{j}\right|-TP_{j}$ and $FN_{j}=\left|{SC}_{i(L_{SCi}==L_{ICj})}\right|-TP_{j}$. By taking the union of all ${IC}_{j}$, we obtain $TP=\;{⋃}_{j=\text{1}}^{M}TP_{j}$, $FP=\;{⋃}_{j=\text{1}}^{M}FP_{j}$, and $FN=\;{⋃}_{j=\text{1}}^{M}FN_{j}$.

When TP, FP and FN are determined, precision and recall are then calculated. As the harmonic mean of precision and recall, FM is an aggregate measure of the inferred cluster’s quality.


$$Precision=\frac{\left|TP\right|}{\;|TP|+|FP|}$$

$$Recall=\frac{\left|TP\right|}{\;|TP|+|FN|}$$

$$FM=\frac{\text{2}*Precision*\;Recall}{Precision+\;Recall}$$

TIC Purity, TIC Retention, TIC Fraction: We defined ${TIC}_{i}$ (i = 1…N*) as the inferred cluster that had the largest number of sequences $L_{SCi}$ from given simulated cluster and reconstructed at least 80% of its family members ${SC}_{i}$. In contrast, noise clusters comprised solely of noise sequences, while any remaining clusters were categorized as mix clusters.

TIC Fraction: The fraction of the target signal within the total signal. A higher value indicates better performance.


$$TIC\;Fraction=\frac{N^{*}}{M}$$

TIC Purity: The proportion of the target signal in the total signal. A higher value indicates better performance.


$$TIC\;Purity=\frac{\left|{TIC}_{i(L==\;L_{{SC}_{i}})}\right|}{\left|{TIC}_{i}\right|}$$

TIC Retention: The retention time of the target signal (normalized to the range of 0–1). A higher value indicates better performance.


$$TIC\;Retention=\frac{\sum_{i\;=\;\text{1}}^{N^{*}}\left|{TIC}_{i(L==\;L_{{SC}_{i}})}\right|}{\sum_{i\;=\;\text{1}}^{N}\left|{SC}_{i}\right|}$$

### Quantification and statistical analysis

Statistical comparisons in Figs 3C-3D and 5C–5F were carried out by the Kruskal-Wallis test, in Figs 4E-4G and 6C–6H were carried out by the two-sided Wilcoxon rank-sum test (*P <= 0.05; **P <= 0.01; ***P <= 0.001). All boxplots showed the lower, median, and upper quartiles of the values. Error bars on the bar plots represent standard deviation of the mean. Scatter plots in Figs 2A-2C, 3A-3B, 4E–4G, and 5H-5I were analyzed by calculating the Spearman correlation coefficients and their corresponding p-values. In addition, the regression analysis used to generate Fig 3E was performed with the glmmTMB package using beta regression. In this analysis, the corresponding p-values were computed from the model summaries. All statistical analyses were performed using R statistical programming language (version 4.4.0). Details of the statistical tests are provided in the corresponding figure legends.

## Supporting information

S1 Table Public paired BCR-seq datasets used in this study. (PDF)

S1 Fig Evaluation of LC consistency based on HC clustering, focusing on clusters of size three. (PDF)

S2 Fig Evaluation of LC features based on HC clustering, focusing on clusters of size four. (PDF)

S3 Fig Correlation between LC V-gene/J-gene consistency scores and sequenceing depth and cluster size. (PDF)

S4 Fig Distributation of HC and LC SHM load between high-consistency clusters in naive and memory B cells. (PDF)

S5 Fig Pre-training performance assessment of BCR-V-BERT models. (PDF)

S6 Fig Evaluation of the heavy and light chain public BCR prediction model. (PDF)

S7 Fig Performance comparison of PubBCRp, antiBERTa-based embeddings, and OLGA generative models for publicness prediction. (PDF)

S8 Fig The distributation of predicted light and heavy chain public scores in Plasma and Unsorted B cell samples. (PDF)

S9 Fig Determination of publicness and SHM cutoffs for filtering pseudo-clonal clusters. (PDF)
