# Supplementary material for: Large-scale paired chain BCR analysis reveals antibody clonal family inference bias and enhances resolution with machine learning
Source: PLoS Comput Biol. 2026 Mar 11;22(3):e1014077. doi: 10.1371/journal.pcbi.1014077 (PMC12998946; doi:10.1371/journal.pcbi.1014077)
Supplement: S1 Table — (PDF) [file pcbi.1014077.s001.pdf]

S1 Table. Public paired BCR-seq datasets used in this study

Dataset	Disease	Number of samples	Number of unique productive sequences	Identifier
Jaffe et al., 2022[1,2]	SARS-CoV-2, CMV, Healthy	94	843~21,839	DOI: 10.25452/figshare.plus.20338177
Ramesh et al, 2020[3]	Multiple-sclerosis	54	169~13,687	BioProject: PRJNA549712 (NCBI SRA/BioProject)
Phad et al., 2022[4]	Healthy	41	1,068~8,478	GenBank: OL450601–OL451038 (NCBI Nucleotide)
Mor et al., 2021[5]	SARS-CoV-2	15	1,584~4,393	BioProject: PRJNA670581 (NCBI SRA/BioProject)
Woodruff et al., 2020	SARS-CoV-2	2	1,534~1,896	BioProject: PRJNA642962 (NCBI SRA/BioProject)
Sokal et al, 2021[7]	SARS-CoV-2	12	1,478~11,158	ArrayExpress: E-MTAB-9995 (EMBL-EBI ArrayExpress)
Eccles et al., 2020[8]	Healthy	1	624~624	BioProject: PRJNA580187 (NCBI SRA/BioProject)

REFERENCES

1. Jaffe, D.B., Shahi, P., Adams, B.A., Chrisman, A.M., Finnegan, P.M., Raman, N., Royall, A.E., Tsai, F., Vollbrecht, T., Reyes, D.S., et al. (2022). Functional antibodies exhibit light chain coherence. Nature *611*, 352–357. https://doi.org/10.1038/s41586-022-05371-z.

2. Jaffe, D.B., Shahi, P., Adams, B.A., Chrisman, A.M., Finnegan, P.M., Raman, N., Royall, A.E., Tsai, F., Vollbrecht, T., Reyes, D.S., et al. (2022). Functional antibodies exhibit light chain coherence. https://doi.org/10.5281/zenodo.6471398.

3. Ramesh, A., Schubert, R.D., Greenfield, A.L., Dandekar, R., Loudermilk, R., Sabatino, J.J., Koelzer, M.T., Tran, E.B., Koshal, K., Kim, K., et al. (2020). A pathogenic and clonally expanded B cell transcriptome in active multiple sclerosis. Proc Natl Acad Sci U S A *117*, 22932–22943. https://doi.org/10.1073/pnas.2008523117.

4. Phad, G.E., Pinto, D., Foglierini, M., Akhmedov, M., Rossi, R.L., Malvicini, E., Cassotta, A., Fregni, C.S., Bruno, L., Sallusto, F., et al. (2022). Clonal structure, stability and dynamics of human memory B cells and circulating plasmablasts. Nat Immunol *23*, 1076–1085. https://doi.org/10.1038/s41590-022-01230-1.

5. Mor, M., Werbner, M., Alter, J., Safra, M., Chomsky, E., Lee, J.C., Hada-Neeman, S., Polonsky, K., Nowell, C.J., Clark, A.E., et al. (2021). Multi-clonal SARS-CoV-2 neutralization by antibodies isolated

from severe COVID-19 convalescent donors. PLOS Pathogens *17*, e1009165. https://doi.org/10.1371/journal.ppat.1009165.

6. Woodruff, M.C., Ramonell, R.P., Nguyen, D.C., Cashman, K.S., Saini, A.S., Haddad, N.S., Ley, A.M., Kyu, S., Howell, J.C., Ozturk, T., et al. (2020). Extrafollicular B cell responses correlate with neutralizing antibodies and morbidity in COVID-19. Nat Immunol *21*, 1506–1516. https://doi.org/10.1038/s41590-020-00814-z.

7. Sokal, A., Chappert, P., Barba-Spaeth, G., Roeser, A., Fourati, S., Azzaoui, I., Vandenberghe, A., Fernandez, I., Meola, A., Bouvier-Alias, M., et al. (2021). Maturation and persistence of the anti-SARS-CoV-2 memory B cell response. Cell *184*, 1201-1213.e14. https://doi.org/10.1016/j.cell.2021.01.050.

8. Eccles, J.D., Turner, R.B., Kirk, N.A., Muehling, L.M., Borish, L., Steinke, J.W., Payne, S.C., Wright, P.W., Thacker, D., Lahtinen, S.J., et al. (2020). T-bet+ Memory B Cells Link to Local Cross-Reactive IgG upon Human Rhinovirus Infection. Cell Reports *30*, 351-366.e7. https://doi.org/10.1016/j.celrep.2019.12.027.
