# Supplementary material for: Large-scale paired chain BCR analysis reveals antibody clonal family inference bias and enhances resolution with machine learning
Source: PLoS Comput Biol. 2026 Mar 11;22(3):e1014077. doi: 10.1371/journal.pcbi.1014077 (PMC12998946; doi:10.1371/journal.pcbi.1014077)
Supplement: S2 Fig — (PDF) [file pcbi.1014077.s003.pdf]

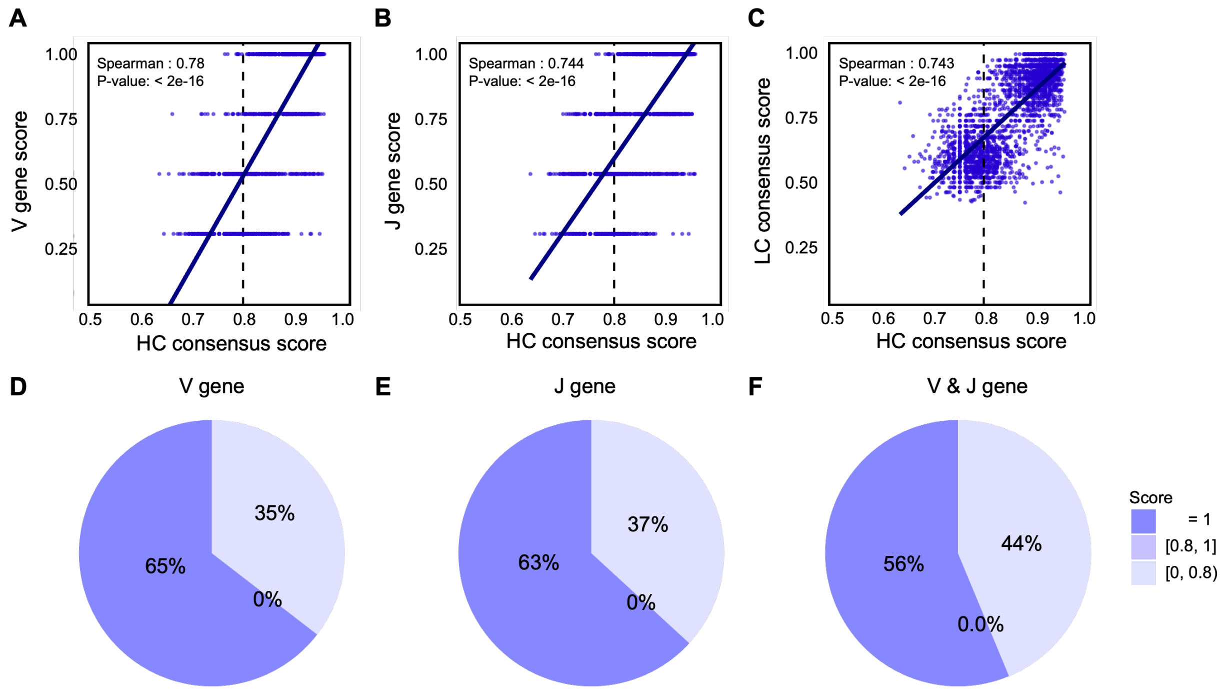

S2 Fig. Evaluation of LC features based on HC clustering, focusing on clusters of size four. (A–C) Scatter plots illustrating the correlation between LC V-gene/J-gene/CDR3 consistency and HC clustering consistency. (D–F) Pie charts of LC consistency in clusters where HC consistency ≥0.8, categorized by V gene, J gene, or both.
