# Supplementary material for: Large-scale paired chain BCR analysis reveals antibody clonal family inference bias and enhances resolution with machine learning
Source: PLoS Comput Biol. 2026 Mar 11;22(3):e1014077. doi: 10.1371/journal.pcbi.1014077 (PMC12998946; doi:10.1371/journal.pcbi.1014077)
Supplement: S3 Fig — (PDF) [file pcbi.1014077.s004.pdf]

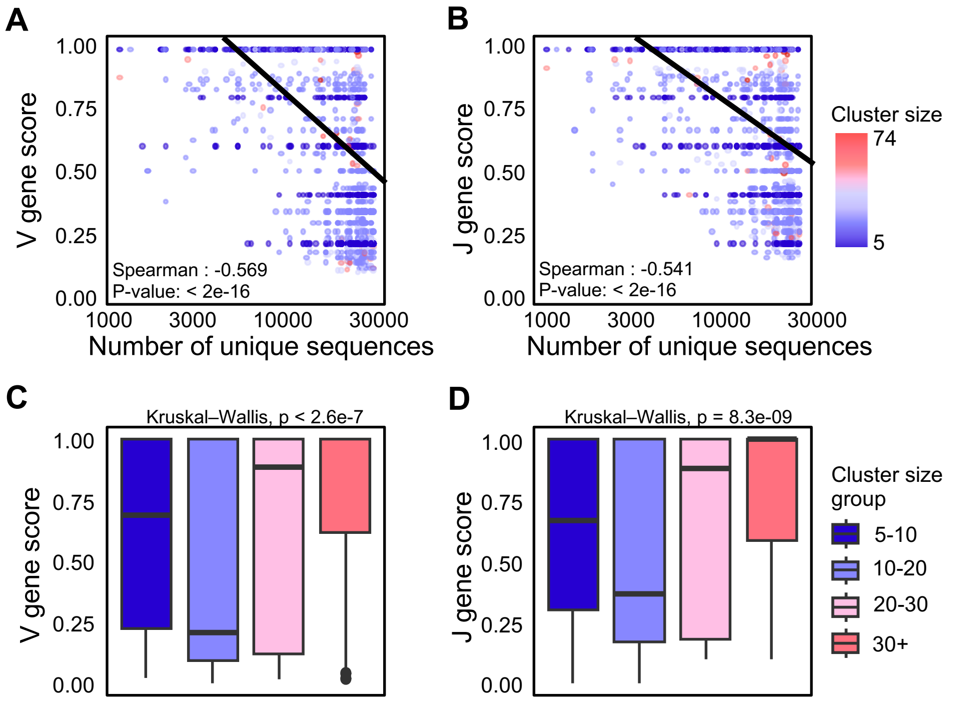

S3 Fig. Correlation between LC V-gene/J-gene consistency scores and sequenceing depth and cluster size. (A-B) Scatter plots showing how sequencing depth and cluster size relate to LC V-gene/J-gene consistency scores, point color indicates cluster size, and lines represent fitted regression trends. (C-D) Box plots of LC V-gene/J-gene consistency score stratified by cluster-size groups. The black horizontal line denotes the median, boxes span the interquartile range, and dots represent outliers.
