# Supplementary material for: Large-scale paired chain BCR analysis reveals antibody clonal family inference bias and enhances resolution with machine learning
Source: PLoS Comput Biol. 2026 Mar 11;22(3):e1014077. doi: 10.1371/journal.pcbi.1014077 (PMC12998946; doi:10.1371/journal.pcbi.1014077)
Supplement: S4 Fig — (PDF) [file pcbi.1014077.s005.pdf]

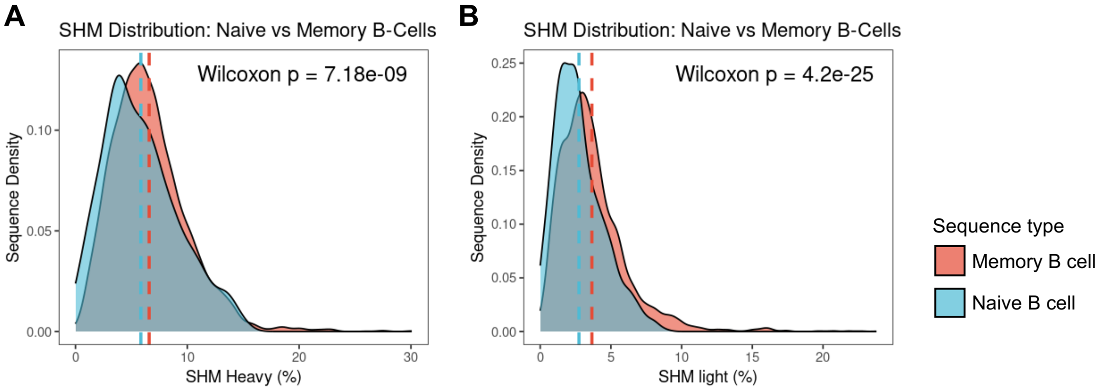

S4 Fig. Distributation of HC and LC SHM load between high-consistency clusters in naive and memory B cells. (A-B) Density plot showing the distribution of heavy-chain (A) and light-chain (B) somatic hypermutation (SHM) levels across high-consistency clusters in naive and memory B cells.
