# Supplementary material for: Large-scale paired chain BCR analysis reveals antibody clonal family inference bias and enhances resolution with machine learning
Source: PLoS Comput Biol. 2026 Mar 11;22(3):e1014077. doi: 10.1371/journal.pcbi.1014077 (PMC12998946; doi:10.1371/journal.pcbi.1014077)
Supplement: S5 Fig — (PDF) [file pcbi.1014077.s006.pdf]

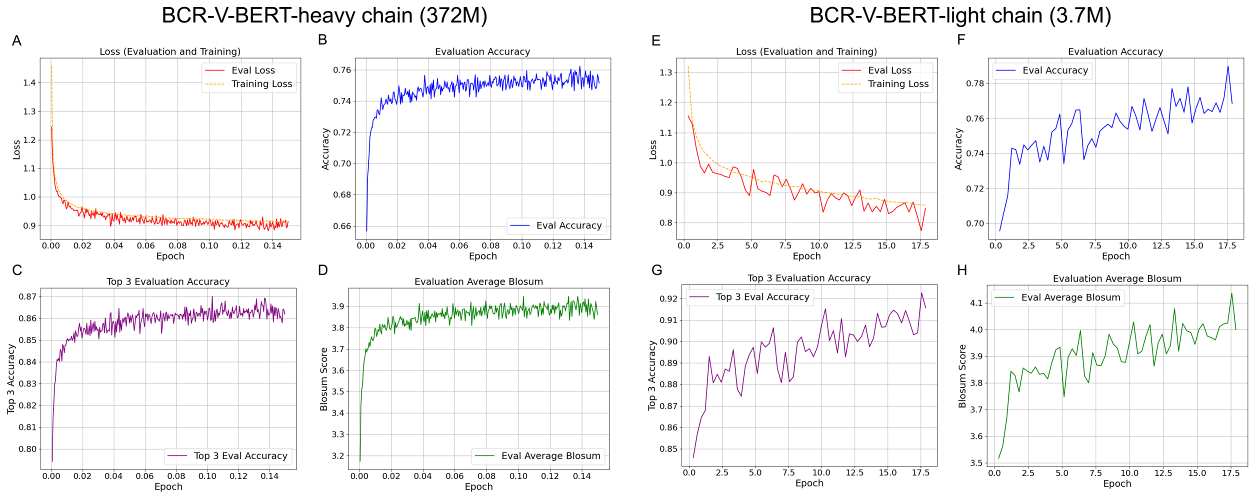

S5 Fig. Pre-training performance assessment of BCR-V-BERT models. The curve plots show the training and testing performance of the heavy (left panel) and light (right panel) chain BCR-V-BERT model during pre-training. The loss (A, E) and Top-1 (B, F), Top-3 (C, G) and BLOSUM (D, H) accuracy curves for both the training and test datasets are presented to evaluate the model's learning progress. The loss is computed using Masked Language Model (MLM) loss, which involves randomly masking parts of the input sequence and training the model to predict the masked tokens. This self-supervised learning objective helps the model to capture contextual relationships between tokens in the sequence. Top-1 accuracy measures the percentage of times the model's top prediction is correct, while Top-3 accuracy considers the correct label within the model's top three predictions. BLOSUM accuracy evaluates the model’s ability to capture biologically relevant sequence similarities using BLOSUM scores.
