# Supplementary material for: Large-scale paired chain BCR analysis reveals antibody clonal family inference bias and enhances resolution with machine learning
Source: PLoS Comput Biol. 2026 Mar 11;22(3):e1014077. doi: 10.1371/journal.pcbi.1014077 (PMC12998946; doi:10.1371/journal.pcbi.1014077)
Supplement: S6 Fig — (PDF) [file pcbi.1014077.s007.pdf]

A

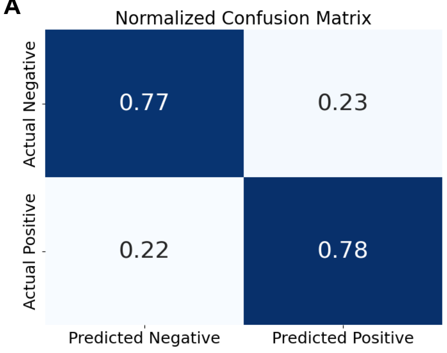

C

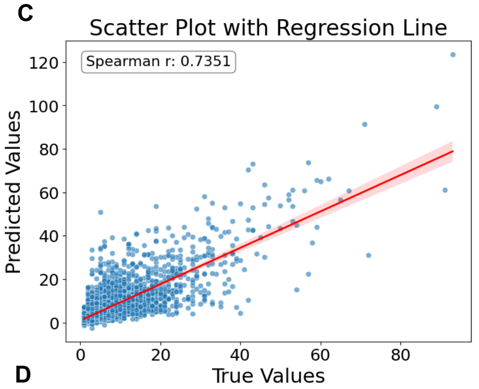

B

Accuracy	0.7734
Specificity	0.7684
Precision	0.7707
Recall	0.7783
F1-Score	0.7745

D

R Square	0.5607
MSE	20.0482
RMSE	4.4775
MAE	2.5040
MAPE	0.5210

S6 Fig. Evaluation of the heavy and light chain public BCR prediction model. (A) Confusion matrix for the heavy chain classification model on the validation dataset, showing the model's performance in classifying heavy chain sequences. (B) Performance metrics for the heavy chain model, including Accuracy, Specificity, Precision, Recall, and F1-score, to assess classification quality. (C) Correlation plot between predicted and true labels in the independent validation set, with Spearman's rho = 0.7887, indicating the strength of the monotonic relationship between predictions and actual values. (D) Regression performance metrics for the model's continuous predictions, including R^2 (R-squared), Mean Squared Error (MSE), Root Mean Squared Error (RMSE), Mean Absolute Error (MAE), and Mean Absolute Percentage Error (MAPE), which quantify the model's predictive accuracy and error.
