# Supplementary material for: Large-scale paired chain BCR analysis reveals antibody clonal family inference bias and enhances resolution with machine learning
Source: PLoS Comput Biol. 2026 Mar 11;22(3):e1014077. doi: 10.1371/journal.pcbi.1014077 (PMC12998946; doi:10.1371/journal.pcbi.1014077)
Supplement: S7 Fig — (PDF) [file pcbi.1014077.s008.pdf]

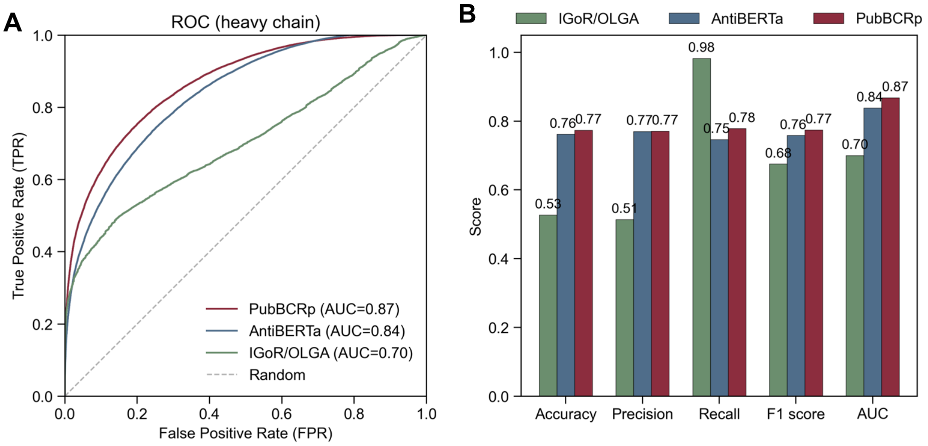

S7 Fig. Performance comparison of PubBCRp, antiBERTa-based embeddings, and OLGA generative models for publicness prediction. (A) ROC curves with AUC values comparing antiBERTa embeddings, the probabilistic generative model OLGA, and PubBCRPredictor on heavy-chain sequences. (B) Summary metrics (Accuracy, Precision, Recall, F1 score, AUC) across all three approaches, showing consistently improved overall performance for PubBCRp.
