# Supplementary material for: Large-scale paired chain BCR analysis reveals antibody clonal family inference bias and enhances resolution with machine learning
Source: PLoS Comput Biol. 2026 Mar 11;22(3):e1014077. doi: 10.1371/journal.pcbi.1014077 (PMC12998946; doi:10.1371/journal.pcbi.1014077)
Supplement: S8 Fig — (PDF) [file pcbi.1014077.s009.pdf]

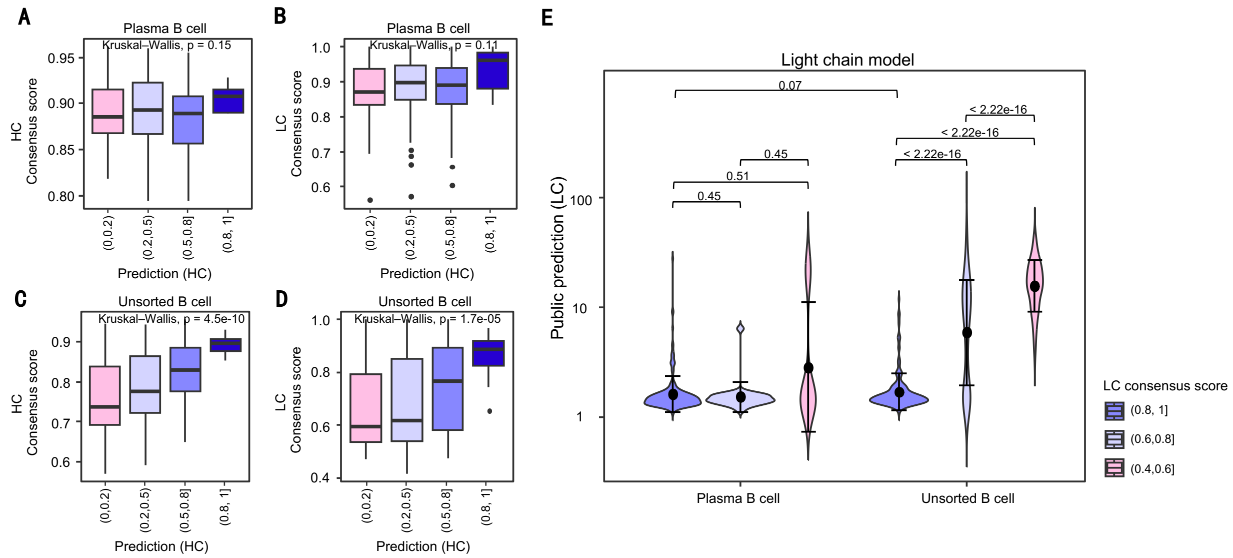


**S8 Fig. The distributation of predicted light and heavy chain public scores in Plasma and Unsorted B cell samples.** Supplementary data corresponding to Figure 5 (C-G). (A-D) Grouping clusters by their publicness scores, we explore how plasma (A, B) and unsorted B (C, D) cells' heavy-chain public score relates to LC and HC consistency. Public socre is calculated as the mean predicted probability from the heavy-chain classification model (range 0-1). Clusters are divided into four bins (1, [0.5, 0.8), [0.2, 0.5), [0, 0.2)). Median values are shown by black lines; points represent outliers. Kruskal-Wallis tests were used for statistical comparisons. (E) Violin plots illustrating how LC consistency groups affect the publicness scores predicted by the light-chain regression model across unsorted and plasma cells. Black dots show means, vertical lines show standard deviations, and colors denote distinct LC groups.
