# Supplementary material for: Large-scale paired chain BCR analysis reveals antibody clonal family inference bias and enhances resolution with machine learning
Source: PLoS Comput Biol. 2026 Mar 11;22(3):e1014077. doi: 10.1371/journal.pcbi.1014077 (PMC12998946; doi:10.1371/journal.pcbi.1014077)
Supplement: S9 Fig — (PDF) [file pcbi.1014077.s010.pdf]

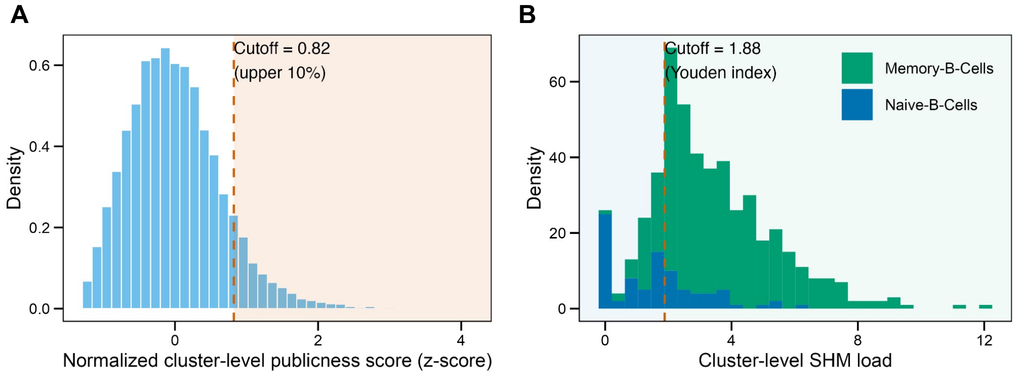

S9 Fig. Determination of publicness and SHM cutoffs for filtering pseudo-clonal clusters. (A) Distribution of cluster-level public scores. Public clusters were identified by z-score normalizing heavy- and light-chain publicness scores, with a data-driven cutoff corresponding to the top 10% of the standardized score distribution. (B) Distribution cluster-level SHM load across naive and memory B-cell clusters. The SHM cutoff was derived from ROC analysis using annotated naive and memory B-cell clusters, corresponds to the maximal Youden index. Public clusters with low SHM were filtered out, while those with elevated SHM were retained.
